# A Review of On-Site Wastewater Treatment Systems in Western Australia from 1997 to 2011

**DOI:** 10.1155/2015/716957

**Published:** 2015-04-19

**Authors:** Maria Gunady, Natalia Shishkina, Henry Tan, Clemencia Rodriguez

**Affiliations:** Department of Health, Government of Western Australia, Grace Vaughan House, 227 Stubbs Terrace, Shenton Park, WA 6008, Australia

## Abstract

On-site wastewater treatment systems (OWTS) are widely used in Western Australia (WA) to treat and dispose of household wastewater in areas where centralized sewerage systems are unavailable. Septic tanks, aerobic treatment units (ATUs), and composting toilets with greywater systems are among the most well established and commonly used OWTS. However, there are concerns that some OWTS installed in WA are either performing below expected standards or failing. Poorly performing OWTS are often attributed to inadequate installation, inadequate maintenance, poor public awareness, insufficient local authority resources, ongoing wastewater management issues, or inadequate adoption of standards, procedures, and guidelines. This paper is to review the installations and failures of OWTS in WA. Recommendations to the Department of Health Western Australia (DOHWA) and Local Government (LG) in regard to management strategies and institutional arrangements of OWTS are also highlighted.

## 1. Introduction

**Figure 1:**
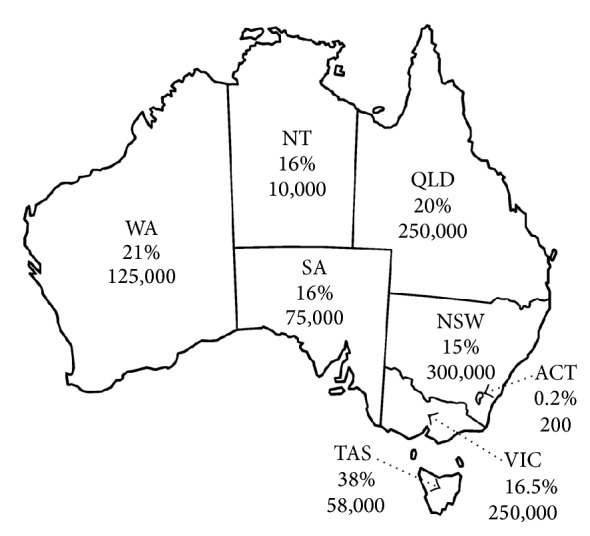
Distribution of on-site wastewater systems in Australia in 2001. Adapted from Beal et al. [9].

On-site wastewater treatment systems (OWTS) are widely utilized to treat and dispose of household wastewater in areas where centralised reticulated sewerage systems are unavailable. Septic tanks, aerobic treatment units (ATUs), and composting toilets with greywater treatment are among the more commonly used OWTS in Australia and elsewhere [1]. In the 1990s it was estimated that approximately 20% of Australian households rely on septic systems for domestic wastewater management [2]. By 2001, it was estimated that more than 1 million OWTS were installed in Australia, with the greatest distribution in New South Wales (300,000), followed by Victoria (250,000) and Queensland (250,000). In Western Australia (WA) itself, there were 125,000 OWTS installed in 2001 [3] (Figure 1). Figure 1 represents the numbers of OWTS installations while the percentages on the figure indicate the proportion of unsewered properties in each state across Australia. These figures are likely to increase along with Australian population growth, the continuous development of rural and unsewered urban regions [4], and the acceptance that OWTS are a cost effective and long-term option for meeting public health and environmental health goals if properly installed, operated, and maintained [4]. Current average in Australia is 2.6 people per household. Assuming each person produces approximately 200 L of wastewater per day a total of approximately 31 tons of nitrogen, 8.4 tons of phosphorus, and 70 × 10^10^ faecal coliform organisms will be discharged to the OWTS [3]. Nutrients and pathogens are released into the environment when OWTS fail and the public health and environmental consequences could be significant considering that OWTS failure in Australia has been reported high, reaching up to 40% [5].

## 2. Impacts of Poorly Performing OWTS

### 2.1. Environmental and Public Health Impacts

Undoubtedly, poorly performing or failing OWTS have economic impacts on the owners and pose environmental impacts due to nutrients that may disrupt ecosystem balance. Furthermore, poorly performed or failed OWTS can contribute to eutrophication of sensitive water bodies and create algae blooms. Failing OWTS are one of the main causes of streams, lakes, rivers, wetlands, and groundwater contamination due to the release of nutrients and pathogens into the environment [5]. Various studies worldwide and in Australia have shown that poorly performing or failing OWTS have resulted in degradation of soil and water quality [3, 6, 7]. Furthermore, effluent from underperformed or failed OWTS may pose a public health risk, particularly to susceptible population group such as children, the elderly, or people with compromised immune system. Contamination of drinking water or recreational waters by failing OWTS may increase concentration of pathogens that may impact on human health [4]. Untreated effluent lying in gutters or pooling in public places provides an environment for the transmission of diseases, such as those listed below:

- gastroenteritis from bacteria such as* Salmonella* or viruses such as rotavirus or protozoa (namely* Giardia*),
- bacterial dysentery,
- typhoid fever from* Salmonella*,
- polio from enterovirus,
- infectious hepatitis (hepatitis A) causing liver disease,
- skin infections from viruses, bacteria, and fungi.

Pooling off effluent creates a nuisance by emitting offensive odours thus affecting the amenity of an area. Spray irrigation of untreated greywater or septic tank effluent can result in aerosol transmission of diseases causing organisms. Irrigation of effluent or greywater water onto food crops can cause diseases such as gastroenteritis or serious life-threatening diseases. Contact with lawn areas recently irrigated with untreated wastewater may also result in skin infections or gastroenteritis via direct or indirect ingestion of organisms [8].

Swimming in water contaminated with effluent may result in skin, ear, and eye infections or gastroenteritis. Similarly, consuming shellfish from effluent contaminated water may result in diseases such as hepatitis A [8].

Most of the public health impacts of poorly performing OWTS are related to pathogens. However the main chemical hazard of poorly performing OWTS is nitrate. Elevated nitrogen levels in drinking water supplies impacted by wastewater contamination may result in irregular haemoglobin levels or* methemoglobinemia* in children [4].

### 2.2. Failures of OWTS

Poor performance or failures of OWTS are often associated with systems not situated and constructed correctly. In particular, areas with low permeability soils and/or high water tables present the greatest challenge in WA. Failure of many onsite systems is generally not due to inherent flaws in system technology but rather due to inappropriate sitting and construction design or their operation and management [10]. Other contributor factors to poor performance or failure of OWTS are poor public awareness, poor local authority guidance, continuous wastewater management issues, poor maintenance procedures, and inadequate governance [2].

The following causes have been identified as the major reasons for OWTS failure [8]:

- lack of area on the properties for the treatment and disposal of wastewater,
- undersized or inadequate wastewater treatment units or absorption trenches,
- poor maintenance, such as failure to desludge septic tanks,
- a change in occupancy from occasional to permanent,
- inappropriate location of absorption trenches, that is, in areas that are poorly drained, subject to flooding or high seasonal water table,
- poor knowledge of operation and maintenance procedures by homeowners/occupiers.

There is an increasing concern with the cumulative environmental impacts and local public health risk of failing or inadequately designed OWTS in unsewered areas [11]. Therefore, at the planning stage, it is important to ensure that inappropriate development does not proceed with an expectation that reticulated sewerage will come at a later date to solve problems being incurred [12].

### 2.3. Legislations and Guidelines

In areas where reticulated sewerage is not available, on-site disposal of wastewater is required. An application to Construct or Install an Apparatus for the Treatment of Sewage in accordance with the* Health (Treatment of Sewage and Disposal of Effluent and Liquid Waste) Regulations 1974* is required to be lodged to the Local Government (LG) in the first instance. If it is intended that the proposed OWTS will handle less than 540 L/day of wastewater and the building to be serviced is a single dwelling, the LG will process the application. If the wastewater volume generated is greater than 540 L/day and the building being serviced is not a single dwelling, the LG will assess the application, prepare a local government report, and forward the application to the DOHWA for approval. The DOHWA is also responsible for the approval of apparatus for sewage treatment within the state and it provides technical information to assist LGs and the community.

These legislations and guidelines for OWTS in unsewered areas in WA are listed and briefly described below.

- * Health Act 1911 (WA)*. Sewage in the act includes sewage from faecal waste, urine from toilets, and water from shower, bath, laundry, and kitchen. It necessitates the collection, treatment, and disposal of sewage and greywater to ensure public health safety [13].
- * Health (Treatment of Sewage and Disposal of Effluent and Liquid Waste) Regulations 1974*. The regulations specifically cover construction, installation, and operation of sewage systems. Applications for the installation of OWTS are only approved in the cases where sewer connections are not available [6].
- * Code of Practice for the Reuse of Greywater in WA.* This Code of Practice supports the long-term reuse of greywater in both sewered and unsewered areas in WA. DOHWA assesses and approves applications when multiple dwellings or commercial premises intend to use greywater for “in-house” end-uses, premises to reuse greywater via spray irrigation, or premises to reuse greywater via subsoil irrigation that produce more than 5,000 L of greywater daily [14].
- * Code of Practice for the Design, Manufacture, Installation, and Operation of Aerobic Treatment Units (ATUs).* This Code of Practice is published under the provisions of the* Health Act 1911 (WA)* and to be used simultaneously with the* Health (Treatment of Sewage and Disposal of Effluent and Liquid Waste) Regulations 1974*. It provides guidance on the design, construction, installation, and operation of ATU to serve single dwellings [15].
- * Code of Practice for Product Approval of On-Site Wastewater Systems in WA.* The Code of Practice specifies the conditions necessary for on-site wastewater systems (OWS) manufacturers who wish to sell their systems to the public or to use the systems for treating and disposing sewage. Applicants are required to demonstrate that the OWS comply with relevant Australian Standards and other regulations through documentations submitted to the DOHWA [16].
- * The DOHWA Is Currently Adopting Standards Australia and Standards New Zealand (AS/NZS) 1547:2000 On-Site Domestic Wastewater Management (AS/NZS 1547.2012).* This standard is performance based and it guides system design based on site and soil assessment, with the aim of achieving sustainable performance. The DOHWA is currently preparing the Code of Practice for On-Site Wastewater Management with endorsement of AS/NZS 1547. It is expected that this Code of Practice will supersede the current codes and regulations.
- * Government Sewerage Policies. *The policies encourage all new developments and subdivisions in WA to connect to reticulated sewerage as the most efficient, socially equitable, and safe method of wastewater disposal [17]. However, in situations where connection to reticulated sewerage is not possible or practicable, the policies set out the on-site wastewater disposal requirements that proposals for developments and subdivisions must meet, in order to obtain the DOHWA approval [17, 18].

## 3. Aim

This paper aims to review the installations and failures of OWTS in WA from 1997 to 2011. Recommendations to the DOHWA and local government in regard to the administration of OWTS are also presented.

## 4. Methods

A literature review related to OWTS legislation, standards, and approval process was undertaken. Following on from this, data on the numbers of OWTS installed in WA was collected from two sources: centralised DOHWA Data Management System and data provided by LG from their own data systems.

As explained above, the LG is responsible for the approval of OWTS producing less than 540 L/day of wastewater while nondomestic and commercial OWTS producing more than 540 L/day are referred to the DOHWA for approval. Inspections of installed OWTS are the responsibility of the LG Environmental Health Officer (EHO). The EHO is required to inspect the OWTS and ensure that installation complies with the approval and conditions issued either by LG or by DOHWA.

The DOHWA database collected the following information: applicant's details, date of application, dwelling address and purpose, type of apparatus installed, and date of inspection. The database was created in 1997 and in order to match the information with the DOHWA database, data from 1997 onwards was requested to LG. Data related to the numbers of OWTS approved by LG was obtained via emails that were distributed in August 2012 to 140 LGs across WA. There were 140 LGs across WA at the time the information was requested [19]. Attached with the email was a Microsoft Excel 2007 spreadsheet containing two tables that LG Environmental Health Officer (EHO) filled out in order to record the number of installations approved. Tables 1 and 2 contain overall numbers of OWTS by year for periods 1997–2011 approved by LG and DOHWA, respectively. All data was checked and analysed in Microsoft Excel 2007.

At the end of the analysed period in 2011, the DOHWA conducted a survey to gather data from various LGs in WA on the primary causes of OWTS failures. The survey in the form of Microsoft Excel 2007 spreadsheet was sent out to 140 LGs across WA. The information collected in the survey included the type of OWTS and the reasons for system failures. Data was then examined, manually screened, and validated to remove any noncompliance. Follow-up interviews via phone calls were conducted to correct any errors in the data.

## 5. Results

### 5.1. Installation

#### 5.1.1. Statistics on OWTS Applications Approved by LGs between 1997 and 2011

Overall, 28 LGs (out of 140) provided data on the numbers of OWTS installations (Table 3). The number represented 20% of the total number of LGs in WA. Nine of the LGs were located within Perth urban areas while 19 others were in the outer suburban areas. Based on the data obtained, more than 12,500 applications were approved between the periods of 1997 to 2011. The highest number of applications was recorded in 2008 with over 1,000 applications, while the lowest was observed in 1997 with less than 500 applications.

#### 5.1.2. Statistics on OWTS Applications Approved by DOHWA between 1997 and 2011

**Figure 2:**
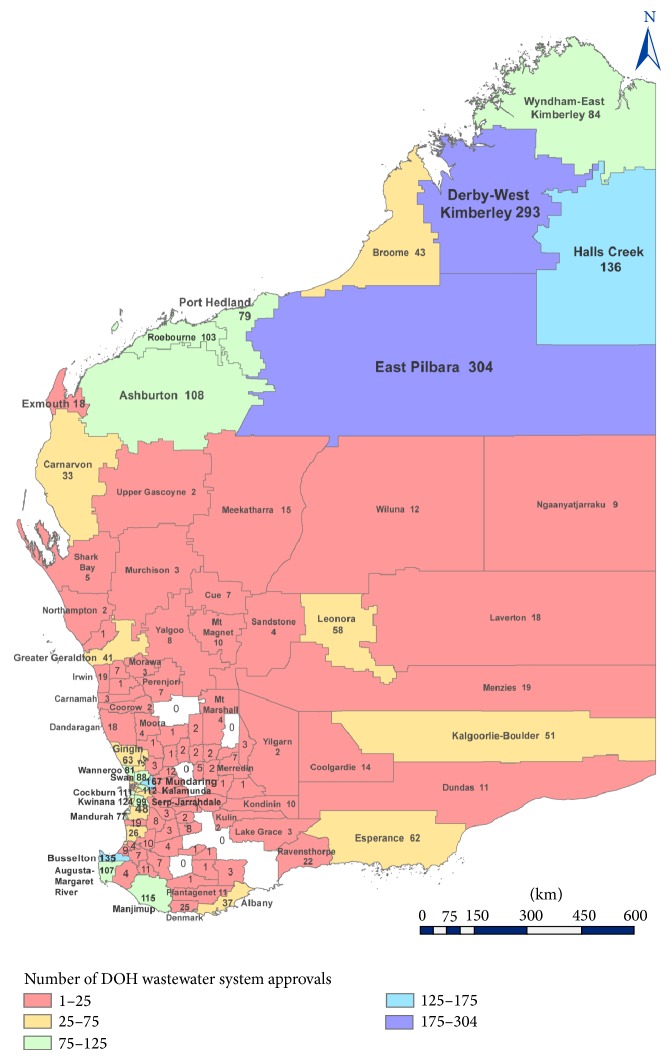
The number of OWTS approved by DOHWA for each LG in WA within periods 1997–2011.

Based on the data collected from the DOHWA database from 1997 to 2011, the DOHWA approved over 3,700 OWTS applications, with an average of 247 applications annually.  Table 4 contains data obtained from various LGs across WA. The highest number of applications was noted in 1999, with over 350 applications whereas the lowest was observed in 1997, with 128 applications. The number of OWTS approved by the DOHWA from January 1997 to December 2011 is shown in Figure 2. East Pilbara and Derby were the two regions with the highest numbers of OWTS approvals, with 304 and 293 approvals, respectively, whereas most of Perth urban areas (e.g., 73 applications in Stirling) recorded relatively low number of installations (Figure 2). This is expected because communities with higher population density in metropolitan and urban areas are more likely to be connected to reticulated sewerage [20].

#### 5.1.3. Number of Installations versus Population Density

The proportions of OWTS installed in WA regional areas are larger than in urban areas. However, the risks of contamination due to failed systems are higher in urban/metro areas due to the increased density of OWTS. Moreover, increasing population density increases the likelihood of release of contaminants such as nitrogen to the environment in OWTS failure [21]. East Pilbara, for example, had the highest number of OWTS approvals between the periods of 1997 and 2011, with a total of 304 installations, whereas City of Stirling has a total of 73 approvals. However, according to the Australian Bureau of Statistics (ABS) [22] the population density of these two LGs is quite different. East Pilbara with an area of over 379,000 km^2^ had a total population of 11,950 in 2011, giving it a population density of 0.031 people per km^2^. On the other hand, City of Stirling had a total population of over 200,000 with a relatively smaller area of 105.2 km^2^, giving it a population density of 1,958 people per km^2^.

#### 5.1.4. Type of OWTS

**Figure 3:**
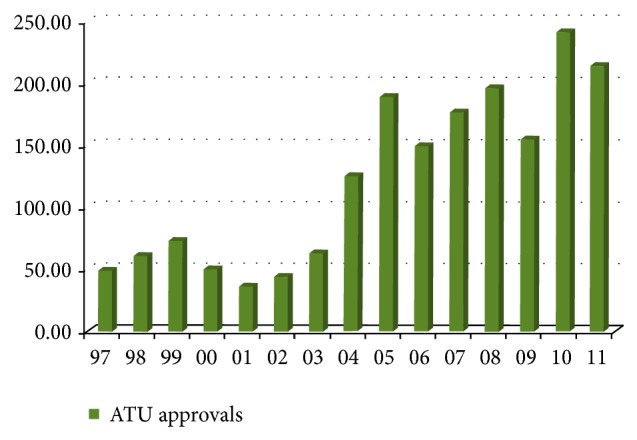
Number of ATU approvals in WA by LG during periods of 1997–2011.

**Figure 4:**
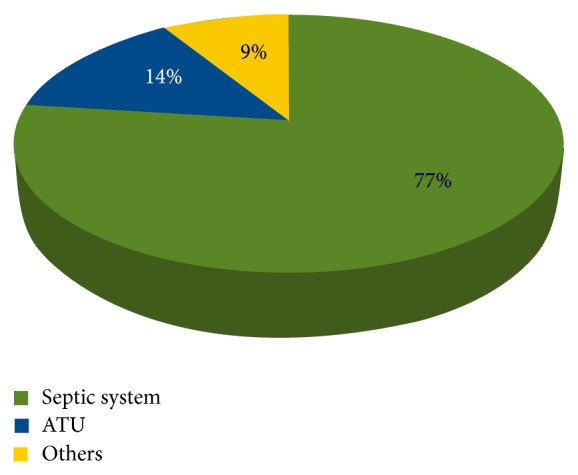
Proportion of OWTS types in WA as approved by LG (1997–2011) (*N* = 12,414).

**Figure 5:**
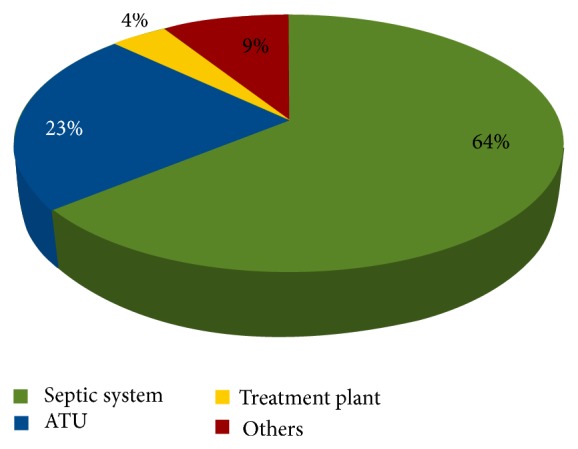
Proportion of OWTS types in WA as approved by DOHWA (1997–2011) (*N* = 1,023).

Data collected from 1997 to 2011 have revealed that conventional septic tanks account for 76.5% and 63.7% of all wastewater systems approved by LG and DOHWA, respectively. Due to its simplicity and affordable cost, septic system remains thus far the most common OWTS. This is despite the fact that ATU is becoming more prevalent. As evident from Figure 3, ATU has become more popular in WA over the last decade due to its ability to produce high quality effluent, as well as its capacity to contain and reuse treated effluent on lawns or garden beds via surface or subsurface irrigation [15]. Figures 4 and 5 indicate that LG and DOHWA have approved more than 1,800 (15%) and over 800 (23%) of ATU installations, respectively, during the study period of 1997 to 2011. The proportions of OWTS types approved by LG and DOHWA are shown in Figures 4 and 5.

There were a total of 9% of OWTS categorized as “other” by LG. More than 80% of the “other” systems were simply described as other without any description or explanation, whereas the remaining 10% corresponds to 2% holding tanks, 4% greywater system, and 4% nutrient removal system. On the other hand, composting was the type described as “other” in the OWTS approved by DOHWA during the study period of 1997 to 2011.

### 5.2. OWTS Failures in WA

As mentioned in Section 4, the DOHWA conducted a survey in 2011 to collect information from various LGs in WA on the major causes of OWTS failures. Out of the 21 LGs that responded back to the survey, 6 were located within Perth metro areas while the remaining 15 LGs were in the outer suburban areas. Based on the data collected, there were a total of 53 OWTS failures, with Shire of Dundas and Shire of Derby/West Kimberley reporting the highest number of failures at 11 and 6 OWTS failures, respectively.

**Figure 6:**
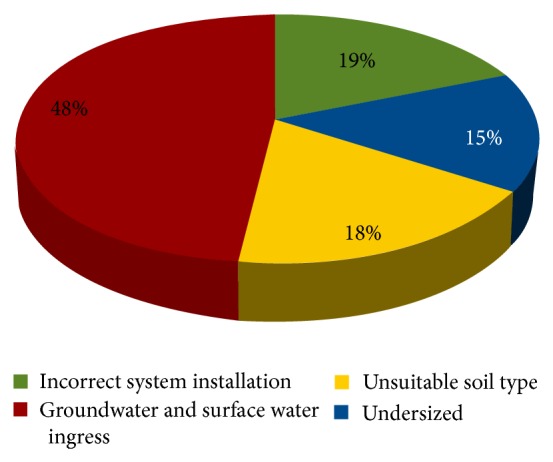
Classification of major OWTS failures in WA (*N* = 53).

The most common reasons for OWTS failures in the survey include groundwater and surface water ingress, systems not installed properly, unsuitable soil type, and undersized systems. Several LGs also mentioned other factors that contributed to OWTS failures, including increase in wastewater volume, root invasion, illegal installation, unauthorised tampering, cross connection to stormwater disposal, undersized systems, and unauthorised materials (such as fats, oils, and yeasts).  Figure 6 presents the classification of major OWTS failures identified by LGs in WA.

## 6. Discussion

Septic systems remain a preferred option for onsite wastewater management in unsewered urban and rural regions of WA. However, the use of other types of OWTS such as ATU and composting toilets with greywater treatment has increased over the years [1]. Given that WA climate is characterised by mild winters and very hot dry summers, there is an increased tendency to improve wastewater quality to achieve fit for purpose nonpotable quality to alleviate the pressure on scarce water resources.

As significant amount of effluent is generated, OWTS have to be installed and monitored properly in order to prevent biological and nutrient contaminants from entering surface water and groundwater [5]. Throughout WA, over 12,500 OWTS applications were approved by 28 LGs during the period of 1997 to 2011, representing 20% of the total number of existing LGs. While 20% of LGs managed to provide statistics of their OWTS approvals, the remaining could not produce any information mainly due to the absence of and difficulties retrieving data. Additionally, more than 3,700 applications were approved through the DOHWA within the same time period, with East Pilbara and Derby being the two regions with the highest number of approvals. As expected, the number of applications was generally higher in regional areas compared to urban areas as most urban areas are connected to centralized sewerage systems [20].

Currently, national and state guidelines are available to ensure adequate construction, installation, and management of OWTS in order to protect public health. However, the survey conducted in 2011 indicated that a number of OWTS are either performing poorly or failing with about 48% of OWTS failures in WA, which is due to groundwater and surface water ingress. Other reasons include incorrect system installation, unsuitable soil type, undersized system, increasing wastewater volume, hydrostatic pressure, high groundwater, root invasion, cross connection to stormwater disposal, and a small percentage of unauthorized tampering. Although technology is improving over time and some OWTS are capable of producing better effluent quality, the problems identified in the DOHWA survey are also reported elsewhere. As such, there is a need to assess site and soil conditions rather than relying solely on technology [23]. Failing or poorly performing systems often lead to various environmental and public health issues [1, 4, 5]. It is common that a slow passage of raw septic tank effluent through 0.5 to 0.6 m of soil results in a high degree of purification. Therefore it is necessary to ensure that there is a minimum of 0.5 to 0.6 m of soil below the base of a trench before high permeable fissured rock, soil, or a groundwater table [24]. In accordance with the AS/NZS 1547:2000, the 600 mm soil absorptive zone is required for filtering, isolating, and absorbing wastewater, microorganisms, nutrients, and partials. Thus, to reduce the potential for OWTS failure due to ground and surface water ingress, implementation of 600 mm absorptive zone is recommended [24].

Population density and number of installed OWTS need to be addressed in more detail. Identifying and comparing areas with high OWTS installations in WA are necessary to ensure that effluents are not negatively impacting human or environmental health. In addition, it is essential to ensure that a fair and consistent approach to dealing with malfunctioning OWTS is taken by LGs under the guidance from the DOHWA.

Inadequate management strategies and the lack of coordinate institutional arrangements in relation to OWTS have been previously reported in Australia [25]. For instance, in a survey of 48 septic tanks, it was found that 72% of dispersal fields were soggy and (by inference) ineffective, 67% required solids removal, 8% needed structural repairs, 6% had insufficient capacity, and 4% were incorrectly sited [5]. Also, a study of OWTS performance in South Australia (SA) indicates that 22% of ATUs contains indicator bacteria at levels higher than the maximum acceptable levels specified in the SA's regulatory guidelines [26].

Collecting data of OWTS installations was conducted by the DOHWA via emails, relying on individual LG across WA to provide data on the number of OWTS installed during the study period. However, as previously mentioned, data regarding the number of approved OWTS applications by LG was only collected from 20% of the existing LGs in WA (28 out of 140). While responses were not expected from many of the metropolitan LGs as they have infill sewerage systems it is acknowledged that response was not received from some LGs with OWTS. A follow-up on the LG that failed to respond to the email sent out by the DOHWA staff enquiring about the numbers of OWTS approvals indicates inadequate number of staff to review the applications or lack of adequate records to respond to the survey. Among those who responded, however, several EHOs found it difficult to complete the task due to lack of simple and efficient methods to collate/retrieve the information.

Several reasons for the lack of available data from LGs include the following.

- Records were not available, due to lack of general file, LG in remote locations, and lack of full time EHOs.
- Records were not easily retrieved as data stored in offsite storage.
- Information on OWTS was not recorded in the early years.
- For some LGs approval, inspection, and ongoing management for OWTS were conducted in a reactive way rather than on a proactive manner.
- EHOs do not have the time to collate information because OWTS are only one part of their several duties.
- Systems described as “other” were not explained or elaborated further.

## 7. Conclusions and Recommendations

Lack of appropriate administration system to record OWTS approvals and installations is a significant drawback in the management of OWTS in WA. Without any feasible management strategies to assess and maintain the performance of OWTS in each region, the resulting environmental and public health risks associated with poor system performance would increase. Hence, there is a need to provide a generic approach for a standardized, common system that will allow each LG to record the number of OWTS applications that have been approved and to store information related to the approvals in a clear and standard record system. Furthermore, there is a need for a multigovernance approach that can address the importance of an effective and robust planning and development of sites. Government agencies need to understand their roles and responsibilities in the management of OWTS when assessing applications for land-use or other developments to ensure that all potential effects arising from such developments are considered prior to approvals. It is important that the site assessment for on-site wastewater management be carried out in the early stage of planning phase. This site assessment procedure should be applied at the rezoning or subdivision stages of the planning process. This will help to develop a management regime for OWTS that can minimize health risks and environmental impacts and enhance OWTS's long-term sustainability.

Based on the findings reported above, to improve water quality from OWTS, the DOHWA should do the following.

- It should provide a standard management tool to all LGs for record of OWTS including GPS location. Registration is the first step in complying with the regulatory requirement. Database should be able to record information on installation, operation, maintenance, and any other inspection requirements particularly for ATUs or systems achieving a secondary treated quality effluent.
- It should implement a soil absorption zone path length of at least 600 mm to reduce the potential for OWTS failure due to ground and surface water ingress.
- It should provide EHOs with on-site wastewater management training based on AS 1547 and current legislations as part of their professional development and an important measure of quality assurance for the industry, government, and general public.
- It should produce educational materials to owners of OWTS to facilitate the understanding of how OWTS work, how to identify signs of systems failures, and why inadequate maintenance can increase the system failures and the risk to humans and the environment.

As recommendations, the LGs should do the following.

- They should implement a risk management program to minimise failures, to rapidly identify and to address failures when problems occur. It is considered that a tolerable rate of system failure is smaller than 5% annually.
- They should implement a planning strategy for OWTS in the early stage of any development to identify the resources that are required to ensure adequate OWTS performance.
- They should implement site inspection strategies that are predominantly risk based, taking account of sensitive receptors, and should be integrated with other regulatory inspections as appropriate.
- They should distribute educational materials produced by the DOHWA or LGs to owners of OWTS and provide advices to applicants on OWTS-related matters when required.
- They should update the register of installed OWTS in each LG jurisdiction and implement an inspection regime to identify legacy sites that will require remediation work. Inspection can initially be targeted at the highest risk areas or OWTS with previous history of failures and complaints.
- In the case of low-risk sites, a variety of nonroutine inspection strategies and in particular proxy (e.g., monitoring of water quality), third party, engagement, and incentive strategies may be implemented. These strategies can involve working closely with home owners or stakeholders to ensure that those who are responsible for OWTS understand how to comply with the regulations and are encouraged to do so.
- They should embark on a series of activities to raise awareness and compliance related to the operation and maintenance of OWTS. Information campaigns may include the communication of international best practice to Water Services Australia (WSA) and specific local guidance to Nongovernment Organizations (NGOs) and homeowners.
- They should conduct follow-up site inspections of approved OWTS that focus specifically on operation and maintenance. Priority should be given to known areas of risk or environmental sensitive areas such as OWTS close to rivers and lakes. Inspections will vary in both type and frequency as the inspection program develops over time.

## Figures and Tables

**Table 1:** OWTS approved by LG (1997–2011).

System type	1997	1998	1999	2000	2001	2002	2003	2004	2005	2006	2007	2008	2009	2010	2011
Septic tank	322	546	782	795	618	630	655	687	556	616	693	753	615	688	626
ATU	49	61	73	50	36	44	63	125	189	149	177	197	155	241	214
Treatment plant	0	0	0	0	0	0	0	0	0	0	1	1	0	0	3
Composting toilet	2	0	0	2	3	2	2	0	1	3	3	1	1	1	2
Others	0	7	7	11	9	16	24	17	35	58	56	61	48	46	41
System not specified	130	90	3	14	60	49	41	54	43	72	18	20	13	11	39

**Table 2:** OWTS approved by DOHWA (1997–2011).

System type	1997	1998	1999	2000	2001	2002	2003	2004	2005	2006	2007	2008	2009	2010	2011
Septic tank	45	43	62	71	61	45	18	14	10	20	31	50	37	35	44
ATU	2	7	7	6	1	3	0	0	3	6	2	10	8	16	14
Treatment plant	0	0	0	0	0	0	0	0	0	0	0	2	1	0	2
Composting toilet	0	0	0	0	1	0	0	0	0	1	0	0	0	0	0
Others	0	0	0	0	0	0	0	1		2	4	3	1	3	4
System not specified	39	38	17	27	31	34	29	14	8	16	17	16	10	16	11

**Table 3:** Type of OWTS approved by LG periods 1997–2011.

Number	Location	System type	Year															Total	Total per LG
			97	98	99	00	01	02	03	04	05	06	07	08	09	10	11		
1	Armadale	Septic tank	62	29	102	104	103	56	55	55	32	48	94	60	114	101	97	1112	1377
		ATU	125								24	16	9	21	23	22	24	264
		Composting toilet														1		1

2	Ashburton	Septic tank													1	10	10	21	72
		ATU													3	10	23	36	
		Composting toilet															1	1	
		Others (holding tanks)													2	4	8	24	

3	Belmont	ATU				1						1						2	
		Others									1			2	1			4	

4	Chittering	Septic tank	5	55	64	48	41	48	58	72	53	71	68	46	45	46	51	771	1164
		ATU	3	8	10	7		3	6	9	7	9	8	13	23	31	20	157	
		Composting toilet	2			2	3		2			3	1					13	
		Others		3	6	9	7	5	17	13	24	34	23	25	24	21	12	223	

5	Cockburn	Septic tank		29	28	33	40	50	55	60	22	25	20	28	16	16	8	430	446
		ATU	1	4	4	1	1	3		2								16	

6	Cue	Septic tank												1	5	3	1	10	10

7	Dalwallinu	Septic tank												1	3	2	1	7	11
		ATU												2	1	1		4	

8	Dandaragan	Septic tank			27	47	31	36	34	44	64	41	33	38	19	16	13	443	447
		ATU												4				4	

9	Dardanup	Septic tank													8	12	13	33	35
		ATU														1	1	2	

10	Derby	Systems not specified	24	11	3	14	10	6	6	8	6	12	18	20	13	11	39	201	201

11	Donnybrook-Balingup	Septic tank											42	53	50	51	59	255	264
		ATU											1	0	4	3	1	9	

12	East Fremantle	Greywater system								1	1	1	1	1	1	1	1	8	8

13	Esperance	Septic tank				77							25	56	40	39	26	263	741
		ATU				1							2	9	6	3	1	22	
		Systems not specified	106	79			50	43	35	46	37	60						456	

		Septic tank	7	7	6	4	2	2	2	2	1					1	1	35	
14	Fremantle	ATU							1	1			1			1	1	5	95
		Others							1		3	2	13	7	14	8	7	55	

15	Gosnells	Septic tank										12	12	17	19	20	19	99	119
		ATU										2	2	4	1	1	3	13	
		Composting toilet											1					1	
		Others										2	3	1				6	

16	Joondalup	Septic tank	14	29	22	17	15	22	12	9	1	2	2	1	1		1	148	148

		Septic tank	77	74	91	73	76	75	66	66	71	70	64	44	45	60	51	1003	
17	Kalamunda	ATU	28	24	23	11	14	13	5	15	18	18	27	19	27	28	20	290	1321
		Composting toilet									1		1		1			3	
		Others		3	1			1		1	1	3	7	2	2	3	1	25	

18	Manjimup	Septic tank	22	68	63	49	33	38	35	26	48	47	42	48	56	42	29	646	665
		ATU		1			2				1		1	4	3	2		14	
		Composting toilet						1									1	2	
		Others									1			2				3	

19	Mundaring	Septic tank	11	93	224	183	113	107	94	29	84	101	115	171	42	125	70	1562	1788
		ATU	2	4	16	15	4	10	12	5	1	4	9	13	5	16	27	143	
		Composting toilet						1										1	
		NRS		1		2	2	8	6	1	3	6	6	6	1	1	2	45	
		Greywater system						2			1	7	3	13	3	4	4	37	

20	Murchison	Septic tank												1				1	1

21	Murray	Septic tank										6	14	38	32	40	23	153	199
		ATU										7	10	10	4	6	9	46	

22	Nedlands	Septic tank					1		2			2	5	2	3	1	4	20	26
		ATU								1	1		1			1		4	
		Others												1	1			2	

23	Northampton	Septic tank													3	4	4	11	11

24	Plantagenet	Septic tank				36	39	47	37	52	41	83	53	61	37	31	34	551	565
		ATU									1	4	1	5			1	12	
		Composting toilet												1				1	
		Others										1						1	

25	Port Hedland	Septic tank											8	3	2	1	3	17	29
		ATU												1		1	4	6	
		Treatment plant											1	1			3	5	
		Others															1	1	

26	Serpentine-Jarrahdale	Septic tank							72	149	48	30	25	16	26	15	47	428	1189
		ATU							24	77	135	88	105	90	55	112	75	761	

27	Stirling	Septic tank	31	38	48	28	21	21	12	3	7	1	1	1	2		1	215	227
		ATU		1	5	1					1					1	2	11	
		Others															1	1	

28	Wanneroo	Septic tank	93	124	107	96	103	128	133	120	84	77	70	67	46	52	60	1360	1377
		ATU												2		1	2	5	
		Others (Ecomax)								1	1	2		1		3	4	12	

**Table 4:** Type of OWTS approved by DOHWA periods 1997–2011.

Number	Location	System type	Year															Total	Total per LG
			97	98	99	00	01	02	03	04	05	06	07	08	09	10	11		
1	Belmont	ATU				1										3		4	25
		Unknown				5	2			1		1		1	4	2	5	21	

2	Cockburn	Septic tank	19	6	9	5	3		7	8	4	3	8	3	3	5	1	84	89
		ATU	1	1	2			1										5	

3	Cue	Septic tank												2				2	3
		Treatment plant													1			1	

4	Dandaragan	Septic tank			27	47	31	36										141	141

5	Derby	Unknown	39	38	17	22	29	34	29	13	8	15	17	15	6	14	9	305	305

6	Donnybrook-Balingup	Unknown															1	1	1

7	Esperance	Septic tank						1	1	1				3	3	1	3	13	24
		ATU												1	2	2	3	8	
		Composting toilet										1						1	
		Others												1			1	2	

8	Fremantle	Septic tank	1	1										1				3	4
		Composting toilet				1												1	

9	Gosnells	Septic tank												1				1	3
		ATU										1					1	2	

10	Joondalup	Septic tank	10	1			1											12	12

11	Kalamunda	Septic tank									3	2	3	2	4	6	4	24	48
		ATU									3	2		5	3	3	3	19	
		Treatment plant															1	1	
		Others											1	1		2		4	

12	Manjimup	Septic tank	6	14	11	7	15	8	8	4	2	4		3				82	92
		ATU	1	1	2	1						2		2				9	
		Composting toilet					1											1	

13	Mundaring	Septic tank	1	1	1	5	3		2		1	9	10	9		5	14	61	76
		ATU		2	1	1		2				1	1			2	2	12	
		Greywater								1								1	
		NRS										2						2	

14	Murchison	Treatment plant												2				2	2

15	Plantagenet	Septic tank										1		1				2	2

16	Port Hedland	Septic tank											9	24	26	13	22	94	114
		ATU											1	1	2	2	4	10	
		Treatment plant															1	1	
		Others											3	1	1	1	3	9	

17	Serpentine-Jarrahdale	Septic tank												1	1	2		4	13
		ATU											1	1	1	4	2	9	

18	Stirling	Septic tank	8	20	14	7	8			1		1	1					60	69
		ATU		3	2	3	1											9	
